# Taxonomic implications of leaf morphology and epidermal anatomy for 14 species of *Gagea* (Liliaceae) from Xinjiang, China

**DOI:** 10.1186/s40529-023-00405-9

**Published:** 2023-11-29

**Authors:** Juan Qiu, Musen Lin, Dunyan Tan

**Affiliations:** Xinjiang Key Laboratory for Ecological Adaptation and Evolution of Extreme Environment Biology, College of Life Sciences, Xinjiang Agricultural University, Ürümqi, 830052 China

**Keywords:** Anatomy, HCA, Leaf epidermal, Taxonomy

## Abstract

**Background:**

Leaf morphology and epidermal characters are important for phylogenetic and taxonomic studies of many plants, but there is currently insufficient data to use them to help distinguish species of *Gagea*, which is a taxonomically difficult genus mainly due to polyploidy and hybridization. Therefore, leaf morphology and epidermal characters of *Gagea* were studied to assess the characters that can be used to elucidate the taxonomy and systematics of 14 species of *Gagea* collected in Xinjiang, China. Using light microscopy (LM), six qualitative and three quantitative leaf epidermal anatomical characters were examined for both the adaxial and abaxial surfaces. Hierarchical cluster analysis (HCA) was employed to reveal the similarities based on leaf morphology and epidermal characters of the investigated species.

**Results:**

Basal leaf of these species can be terete or flat, and it is triangle, flat, or circular in transverse section. Anticlinal wall patterns of the leaf epidermal cells were straight and sinuous, and only three species had epidermal hairs. Shape of long cells varies, ranging from quadrangular to irregular. HCA revealed that the 14 species could be divided into two groups. Group A was arranged into three subgroups (A1, A2 and A3), based on the Euclidean distance of 6.96. Subgroup A1 consisted of three species with indumentum; subgroup A2 had four species with sinuous type anticlinal walls; and subgroup A3 comprised of two species with a fistulose basal leaf. Group B included five species with short cells.

**Conclusions:**

Leaf morphology and epidermal characters did not differ significantly among populations of the same species in *Gagea*, whereas they differ significantly among species. Thus, leaf morphology and epidermal characters provide diagnostic information for differentiating *G. nigra* and *G. filiformis; G. altaica, G. jensii* and *G. alberti*, which are morphologically similar species.

**Supplementary Information:**

The online version contains supplementary material available at 10.1186/s40529-023-00405-9.

## Background

Leaves are crucial vegetative organs that mostly are exposed to aerial conditions. They embody the adaptive survival strategies of plants during the long-term evolution of species (Wang et al. 2021). The use of leaf morphological and epidermal characters has long been recognized as an important tool in species taxonomy (Deng et al. 2017; Filartiga et al. 2022; Khan et al. 2022).

The morphological characters of plant leaves serve as a useful instrument for infrageneric classification and species circumscription, and sometimes they also provide significant information regarding the phylogeny and taxonomy of closely related species (Stace 1969; Baranova 1992; Meng et al. 2016; Ullah et al. 2018; Shaheen et al. 2021; Bashir et al. 2020). High intraspecific consistency may be shown by foliar epidermal characters, although diversity is seen at the interspecific and higher taxonomic levels (Wang et al. 2015; Mohtashamian et al. 2017). Therefore, leaf epidermal features, such as epidermal cell shape, stomatal type, size and distribution, and trichome type and distribution, have played an essential role in distinguishing those families and genera where identification is complicated, such as Salicaceae (Ghahremaninejad et al. 2012), Caryophyllaceae (Ullah et al. 2018), Fabaceae (Silva et al. 2018; Shaheen et al. 2021; Zhao et al. 2022), Lamiaceae (Gul et al. 2019) and Poaceae (Khan et al. 2017).

*Gagea* Salisb. a genus of Liliaceae and tribe Tulipeae (Patterson and Givnish 2022; Peruzzi 2016), is widely distributed from the Mediterranean region throughout Europe and Asia (Zarrei et al. 2007; Peterson et al. 2008, 2019), and plants grow in dry and grasslands, rocky slopes, alpine meadows, open shrublands, and deciduous forest understories (Chen and Turland 2000; Peruzzi 2016; Peterson et al. 2019; Kurbaniyazova et al. 2022). The western Pamir-Alai (97 species) and western Tien-Shan Mountains (65 species) are diversification centers of *Gagea* (Peterson et al. 2011). Due to the wide variety of *Gagea* species, the short, ephemeral growth phase, and complex morphological characters, e.g. an overlap in primitive and advanced morphological characters (the formation of subterranean organs and the number of ground leaves) (Levichev 1999; Peterson et al. 2004), it is difficult to distinguish/identify similar species. Previous studies have investigated different aspects of *Gagea*, including bulb structure (Levichev 1999), morphology and ontogeny (Levichev 2001, 2006; Tison et al. 2013), classical cytotaxonomy (Peruzzi 2003, 2008, 2012; Peruzzi and Aquaro 2005), embryology (Greilhuber et al. 2000; Caparelli et al. 2006), pollen morphology (Kosenko 1999; Zarrei and Zarre 2005; Hu et al. 2021; Sezer and Yildiz 2021; Lin et al. 2023), and molecular systematics (Peterson et al. 2004, 2008, 2016; Peruzzi et al. 2008a, b; Zarrei et al. 2009).

Leaf epidermal morphology in *Gagea* has reported for only 11 species (Elwan 2008; Wang et al. 2013), while anatomical features of the basal leaf has been examined in 22 species (Zarrei et al. 2010). Even at the sectional level, examining the anatomy of the basal leaf and shoot structure in *Gagea* species, provides key taxonomic insights, with crucial characteristics such as the transverse section contour of the leaf, the number of vascular bundles, and the degree of enrichment in the pith providing profound insights for taxonomic research (Zarrei et al. 2010; Levichev 2013). However, due to the low number of species from China, Egypt or Sweden for which epidermal anatomical studies have been conducted, it has not been possible to make intraspecific and interspecific comparisons and thus to explore their taxonomic significance. Thus, we utilized multiple samples of *Gagea* from Xinjiang, China, to investigate leaf and their epidermal morphology by light microscopy (LM), and then we compared the leaf epidermal characters among populations and species to assess their taxonomic significance. Regarding certain species with similar macro-morphology, such as *G. nigra* and *G. filiformis* from the sect. *Minimae*, Peterson et al. (2011) clearly distinguished *G. nigra* from *G. filiformis* using morphological and molecular evidence. Our objective was to confirm this differentiation through micro-morphology. Similarly, within the sect. *Plecostigma*, *G. altaica*, *G. jensii*, and *G. alberti* are grouped together, where it was observed that the morphological similarities between *G. jensii* and *G. alberti*. Our aim was to determine whether the micro-morphology could be used to distinguish between these species.

## Methods

Leaves of the 14 *Gagea* species were collected from plants growing various sites and habitats in Xinjiang, China, and the number of populations for each species ranged from one to six with at least five populations for widespread species (such as *G. bulbifera*, *G. fedtschenkoana* and *G. nigra*) (Table S1; in total 50 populations of *Gagea* were sampled). If there were no differences in leaf morphology and epidermal characters between populations of a species, one population was randomly selected as representative of the species. If populations of a species differed, all populations of the species were studied individually. After collection, the species were identified by comparing their morphological characters to those listed in the *Flora of China* (Chen and Turland 2000); Peterson et al. (2011). The scientific names of species were standardized according to Plants of the World Online (https://powo.science.kew.org).

### Basal leaf materials

In each natural population for a species, a mature and healthy basal leaf was collected from 20 individuals. While in the field, half of each fresh basal leaf was preserved in FAA solution (70% ethyl alcohol, 40% formalin and 40% glacial acetic acid), and the other half was taken back to the laboratory to observe basal leaf size, shape, and state and to photograph a transverse section of the middle portion of the basal leaf with a Nikon SMZ-25 (SMZ25, Japan). The herbarium of Xinjiang Agricultural University (XJA) contains the voucher material for the 14 species of *Gagea*, as listed in Table 1.

**Table 1:** The list of plant materials and voucher specimens used for leaf morphology and leaf epidermal studies of 14 *Gagea* species from Xinjiang, China

Species	Section (Peterson et al. 2016)	Locality	Coordinate	Altitude	Collection Date	Voucher
*Gagea alberti* Regel	*Plecostigm*a	Yining County, Xinjiang, China	43.724521°N, 82.070256°E	1033 m	22 April 2022	J.Qiu & J.L.Li L-062 (XJA)
*G. altaica* Schischk. & Sumnev.	*Plecostigm*a	Fuyun County, Xinjiang, China	46.830949°N, 88.791038°E	703 m	16 April 2021	J.Qiu & M.S.Lin L-009 (XJA)
*G. jensii* Levichev & Schnittler	*Plecostigm*a	Ürümqi City, Xinjiang, China	43.783443°N, 87.544818°E	1002 m	8 April 2021	J.Qiu & M.S.Lin L-005 (XJA)
*G. bulbifera* (Pall.) Salisb.	*Bulbiferae*	Ürümqi City, Xinjiang, China	43.782985°N, 87.544763°E	1003 m	20 April 2021	J.Qiu & M.S.Lin L-014 (XJA)
*G. jaeschkei* Pascher	*Bulbiferae*	Qapqal County, Xinjiang, China	43.411647°N, 81.040648°E	2929 m	17 July 2021	J.Qiu & M.S.Lin L-30 (XJA)
*G. stepposa* L.Z.Shue	*Bulbiferae*	Hutubi County, Xinjiang, China	43.821214°N, 86.429608°E	1160 m	20 April 2022	J.Qiu & J.L.Li L-040 (XJA)
*G. filiformis* (Ledeb.) Kar. & Kir.	*Minimae*	Yuming County, Xinjiang, China	45.842106°N, 82.525295°E	1676 m	28 April 2021	J.Qiu & D.Y.Tan Yu-002 (XJA)
*G. granulosa* Turcz.	*Minimae*	Yuming County, Xinjiang, China	46.191895°N, 82.936688°E	709 m	28 April 2021	J.Qiu & D.Y.Tan Yu-004 (XJA)
*G. nigra* L.Z.Shue	*Minimae*	Yuming County, Xinjiang, China	45.842106°N, 82.525295°E	1676 m	28 April 2021	J.Qiu & D.Y.Tan Yu-001 (XJA)
*G. fragifera* (Vill.) E.Bayer & G.López	*Didymobulbos*	Qinghe County, Xinjiang, China	46.781335°N,90.887534°E	2662 m	6 June 2021	J.Qiu & M.S.Lin L-017 (XJA)
*G. tenera* Pascher	*Didymobulbos*	Nilka County, Xinjiang, China	43.724538°N, 82.070252°E	1033 m	22 April 2022	J.Qiu & J.L.Li L-041 (XJA)
*G. divaricata* Regel	*Platyspermum*	Fuhai County, Xinjiang, China	45.053634°N, 88.398225°E	712 m	17 April 2021	J.Qiu & M.S.Lin L-010 (XJA)
*G. fedtschenkoana* Pascher	*Gagea*	Burqin County, Xinjiang, China	48.504169°N,87.138269°E	1441 m	9 June 2021	J.Qiu & M.S.Lin L-022 (XJA)
*G. kunawurensis* (Royle) Greuter	*Dschungaricae*	Ürümqi City, Xinjiang, China	43.785813°N, 87.545323°E	997 m	20 April 2021	J.Qiu & M.S.Lin L-015 (XJA)

### Leaf epidermal slides observation using light microscope

For isolating the leaf epidermal layer, we used the method of Wen (1995) with some adjustments. Leaves were removed from the FAA fixative and a 1 cm^2^ area was taken from it at the middle of the leaf near the midvein. To remove the adaxial and abaxial epidermal layers, the leaf tissue was soaked in a 50% NaClO solution, washed with water and then the two surface layers were torn off and placed on separate slides. Leaf epidermal tissue was stained in a solution of 1% saffron (50% ethyl alcohol) for 2 hours, dehydrated in an ethanol series, and placed in xylene, which made the tissue transparent, and finally sealed with a cover slip using Canadian balsam.

All slides were labeled, including the voucher number. Five slides of the adaxial and abaxial leaf surface were prepared for each specimen collected in the field, and they were examined with a Nikon Eclipse (80i, Japan) microscope by using an objective lens (at 10× magnification). For each slide, qualitative and quantitative data were recorded. For each slide, qualitative and quantitative characters were recorded; a total of 20 characters for each slide. The five qualitative characters observed were: pattern of anticlinal walls (Ant), short cells (S) present or absent, indumentum (Ind), shape of long cells (Shape) and stomatal type, and four quantitative characters were: long cells size (L), stomata size (St), stomatal index (SI) and density of epidermal cell were observed. Terminology for the description of epidermal cells and stomata follows Barthlott et al. (1998) and Carpenter (2005). Long cells were defined as having a width: length ratio > 3, whereas short cells had a width: length ratio < 3. SI was calculated using the following formula: SI = [S/S + E] × 100, where S = number of stomata per unit area; E = number of epidermal cells per unit area.

### Data analysis

SPSS program version 26 was used to test for normality and homogeneity of the data for leaf epidermal characters to satisfy the requirements of one-way analysis of variance (ANOVA). The differences among different populations of the same species were analyzed by a one-way ANOVA. Differences among species were determined by the non-parametric Kruskal-Wallis test.

Hierarchical cluster analysis (HCA) was employed to reveal similarities among 14 species of *Gagea* in leaf morphology and epidermal characters, as well as taxonomic relationships. Quantitative characters are represented by mean ± standard error (Mean ± SE), whereas qualitative characters were given a specific number in the data matrix. Six qualitative (pattern of anticlinal walls, short cells present or absent, indumentum, shape of long cells, transverse section of basal leaf, cauline leaves present or absent) and three quantitative (long cells size, stomata size and stomatal index) leaf morphology and epidermal characters were evaluated in the comparative analysis for their value in distinguishing the studied *Gagea* species. HCA based on Euclidean distance after normalizing the original data, and the data were clustered using Ward’s method in Origin 2021 software to reflect similarities and differences and achieve data visualization (Ye et al. 2015).

## Results

The observations of leaf morphology and epidermal characters revealed that while qualitative characters remained consistent across various populations, continuous modifications were observed in the quantitative aspects of leaf morphology and epidermal characters. However, there were differences in qualitative leaf morphology and epidermal characters among species and quantitative characters were intermittently distributed among species.

**Fig. 1:**
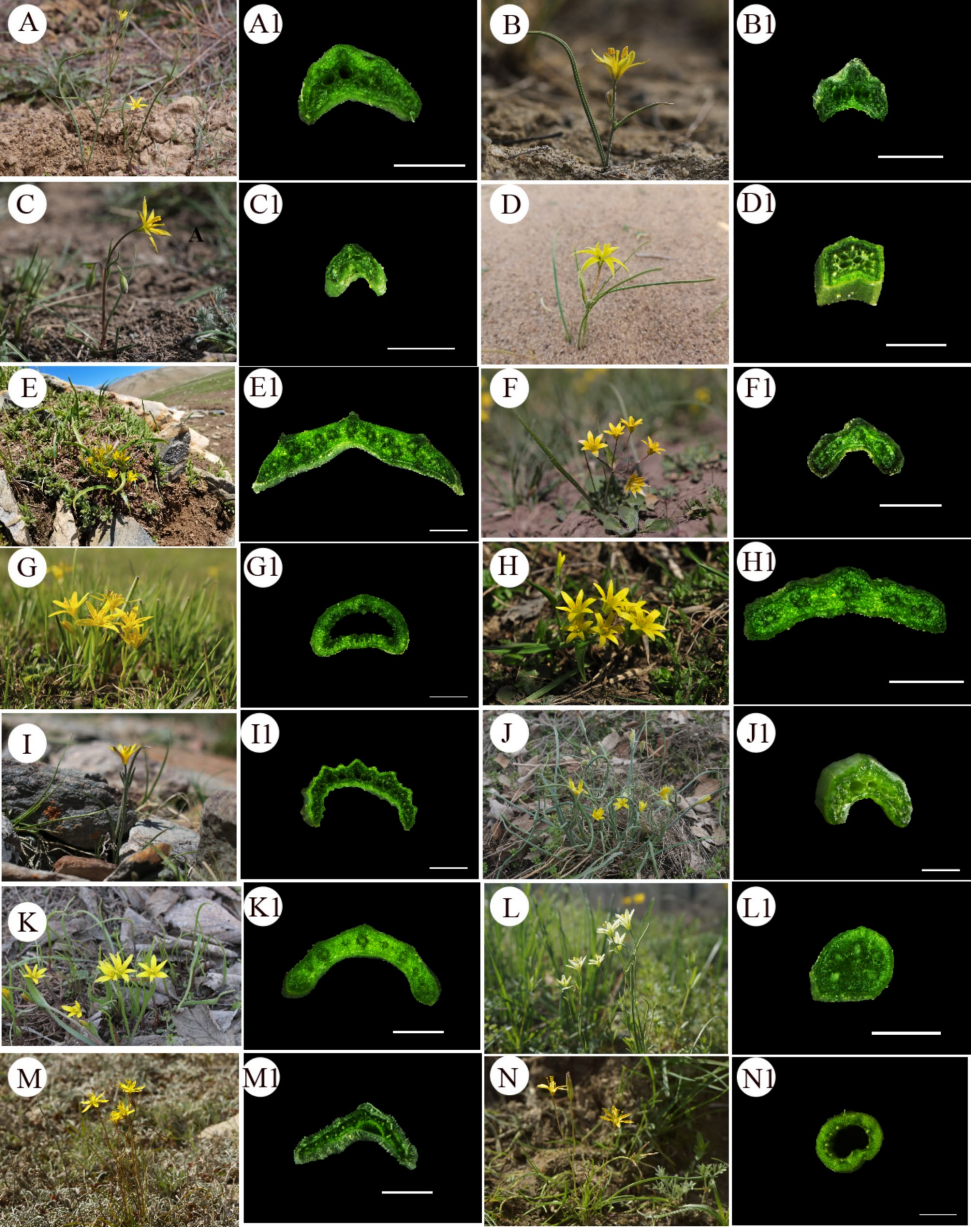
Individual plant and transverse section of basal leaf of 14 species of *Gagea* from Xinjiang, China. (**A-A1**: *Gagea alberti*; **B-B1**: *G. altaica*; **C-C1**: *G. bulbifera*; **D-D1**: *G. divaricata*; **E-E1**: *G. fedtschenkoana*; **F-F1**: *G. filiformis*; **G-G1**: *G. fragifera*; **H-H1**: *G. granulosa*; **I-I1**: *G. jaeschkei*; **J-J1**: *G. jensii*; **K-K1**: *G. nigra*; **L-L1**: *G. kunawurensis*; **M-M1**: *G. stepposa*; **N-N1**: *G. tenera*). Scale bars: 1 mm

**Fig. 2:**
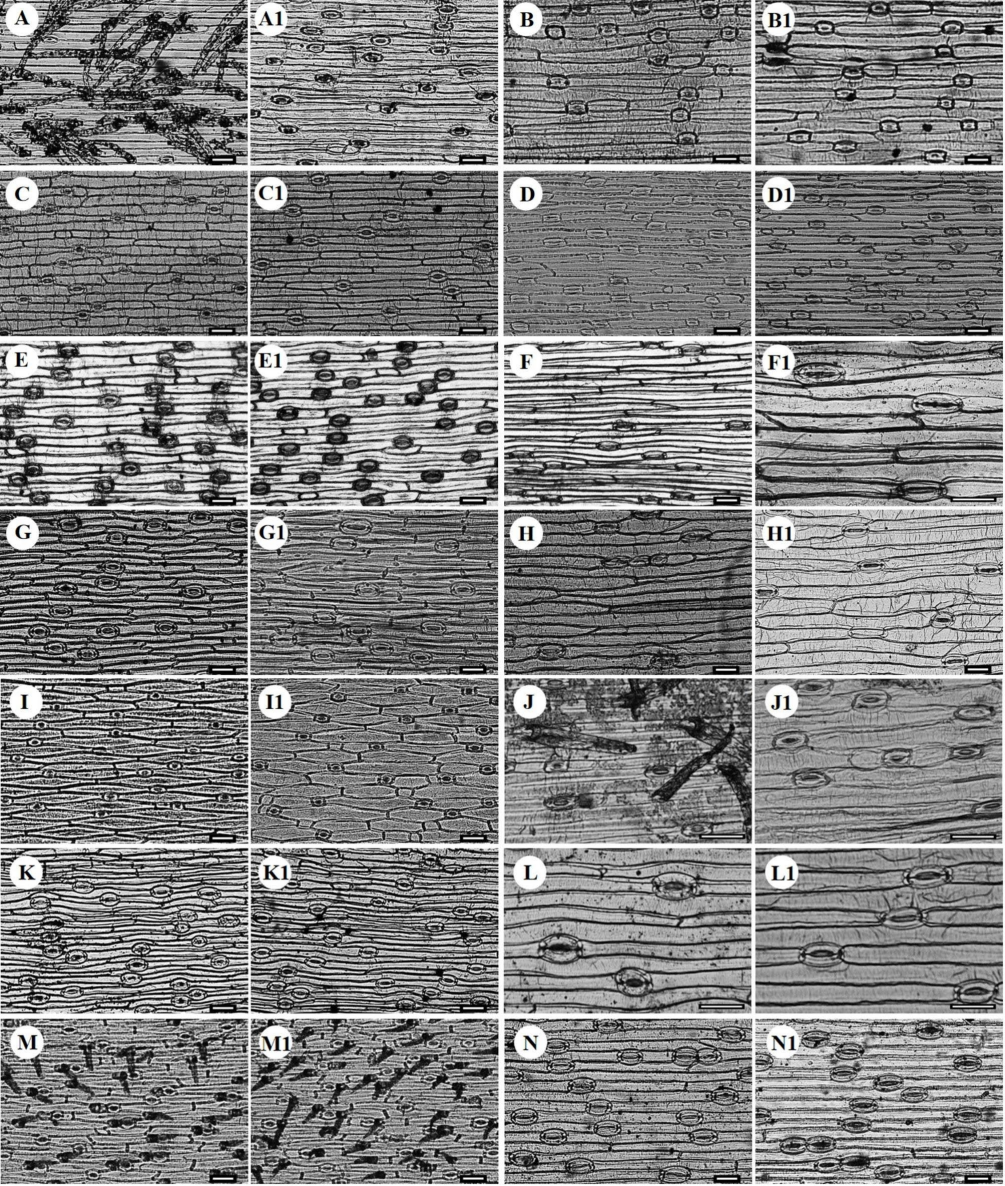
Epidermal morphology of adaxial (A-N) and abaxial (A1-N1) surfaces of basal leaf in 14 species of *Gagea* from Xinjiang, China under light microscope (**A-A1**: *Gagea alberti*; **B-B1**: *G. altaica*; **C-C1**: *G. bulbifera*; **D-D1**: *G. divaricata*; **E-E1**: *G. fedtschenkoana*; **F-F1**: *G. filiformis*; **G-G1**: *G. fragifera*; **H-H1**: *G. granulosa*; **I-I1**: *G. jaeschkei*; **J-J1**: *G. jensii*; **K-K1**: *G. nigra*; **L-L1**: *G. kunawurensis*; **M-M1**: *G. stepposa*; **N-N1**: *G. tenera*). Scale bars: 100 μm

Basal leaf morphology and epidermal characters was studied for 14 species of *Gagea* (Tables 2, 3 and 4). Individual plants and a transverse section of a basal leaf are presented in Fig. 1, and selected LM micrographs of the adaxial and abaxial leaf surface are shown in Fig. 2.

**Table 2:** Morphological characters of leaves of 14 species of *Gagea* from Xinjiang, China (n = 10)

Species	Section (Peterson et al. 2016)	Basal leaf shape	Basal leaf state	Cauline leaves	Basal leaf transverse section	Basal leaf length (mm) Mean ± SE	Basal leaf width (mm) Mean ± SE
*Gagae alberti*	*Plecostigm*a	Flat	Solid	Present	Triangle	156.70 ± 9.45	1.78 ± 0.10
*G. altaica*	*Plecostigm*a	Terete	Solid	Present	Triangle	74.89 ± 4.45	0.92 ± 0.05
*G. jensii*	*Plecostigm*a	Flat	Solid	Present	Triangle	189.76 ± 15.49	2.01 ± 0.14
*G. bulbifera*	*Bulbiferae*	Terete	Solid	Present	Triangle	54.12 ± 3.67	0.70 ± 0.04
*G. jaeschkei*	*Bulbiferae*	Flat	Solid	Present	Flat	154.01 ± 13.17	2.51 ± 0.19
*G. stepposa*	*Bulbiferae*	Flat	Solid	Present	Triangle	84.53 ± 2.77	0.96 ± 0.09
*G. divaricata*	*Platyspermum*	Terete	Solid	Absent	Triangle	132.93 ± 4.98	1.25 ± 0.04
*G. fedtschenkoana*	*Gagea*	Flat	Solid	Absent	Flat	131.20 ± 7.25	3.94 ± 0.24
*G. fragifera*	*Didymobulbos*	Flat	Fistulose	Absent	Circular	177.05 ± 9.01	2.49 ± 0.23
*G. tenera*	*Didymobulbos*	Terete	Fistulose	Present	Circular	73.06 ± 7.18	1.91 ± 0.12
*G. filiformis*	*Minimae*	Terete	Solid	Absent	Triangle	100.45 ± 4.57	1.93 ± 0.09
*G. granulosa*	*Minimae*	Flat	Solid	Absent	Flat	144.98 ± 5.13	4.65 ± 0.22
*G. nigra*	*Minimae*	Flat	Solid	Absent	Flat	116.30 ± 2.95	3.26 ± 0.10
*G. kunawurensis*	*Dschungaricae*	Terete	Solid	Present	Circular	130.15 ± 4.85	1.02 ± 0.04

**Table 3:** Qualitative character states of leaf epidermis in 14 species of *Gagea* from Xinjiang, China

Species	Pattern of anticlinal walls		Long cells shape		Stomatal type		Indumentum		Short cells	
	Ad	Ab	Ad	Ab	Ad	Ab	Ad	Ab	Ad	Ad
*Gagea alberti*	Straight	Straight	Rectangular	Rectangular	Paracytic	Paracytic	Present	Absent	Present	Present
*G. altaica*	Sinuous	Sinuous	Rectangular	Rectangular	Paracytic	Paracytic	Absent	Absent	Present	Present
*G. bulbifera*	Sinuous	Sinuous	Rhomboid	Rhomboid	Paracytic	Paracytic	Absent	Absent	Present	Present
*G. divaricata*	Straight	Straight	Rhomboid	Rhomboid	Paracytic	Paracytic	Absent	Absent	Present	Present
*G. fedtschenkoana*	Sinuous	Sinuous	Irregular	Irregular	Paracytic	Paracytic	Absent	Absent	Present	Present
*G. filiformis*	Sinuous	Sinuous	Irregular	Irregular	Paracytic	Paracytic	Absent	Absent	Absent	Absent
*G. fragifera*	Straight	Straight	Rhomboid	Rhomboid	Paracytic	Paracytic	Absent	Absent	Absent	Absent
*G. granulosa*	Sinuous	Sinuous	Rectangular	Rectangular	Paracytic	Paracytic	Absent	Absent	Present	Present
*G. jaeschkei*	Straight	Straight	Rhomboid	Rhomboid	Paracytic	Paracytic	Absent	Absent	Present	Present
*G. jensii*	Straight	Straight	Rectangular	Rectangular	Paracytic	Paracytic	Present	Absent	Present	Present
*G. nigra*	Sinuous	Sinuous	Irregular	Irregular	Paracytic	Paracytic	Absent	Absent	Present	Present
*G. kunawurensis*	Sinuous	Sinuous	Irregular	Irregular	Paracytic	Paracytic	Absent	Absent	Absent	Absent
*G. stepposa*	Straight	Straight	Rhomboid	Rhomboid	Paracytic	Paracytic	Present	Present	Absent	Absent
*G. tenera*	Straight	Straight	Rectangular	Rectangular	Paracytic	Paracytic	Absent	Absent	Absent	Absent

*Note*: Ad: adaxial; Ab: abaxial

**Table 4:** Quantitative character of leaf epidermis in 14 species of *Gagea* from Xinjiang, China (Mean ± SE) (n = 20)

Species	Long cells (L × W) (um)		Stomata size (L × W) (um)		Stomatal index (%)			Density of epidermal cell (number/mm^2^)		
	Ad	Ab	Ad	Ab	Ad	Ab		Ad	Ab	
*Gagea alberti*	469.64 ± 6.63 × 21.52 ± 0.92	497.93 ± 42.94 × 25.38 ± 0.29	57.26 ± 1.37 × 29.48 ± 0.38	51.02 ± 3.83 × 26.34 ± 0.47	29.02	28.51		51.98 ± 3.39	52.96 ± 6.54	
*G. altaica*	406.54 ± 22.71 × 23.51 ± 2.74	400.08 ± 31.34 × 28.60 ± 2.03	48.16 ± 1.07 × 27.63 ± 0.95	59.25 ± 1.36 × 31.47 ± 1.01	31.17	37.26		59.95 ± 4.68	69.58 ± 5.43	
*G. bulbifera*	277.75 ± 11.03 × 19.92 ± 0.79	288.32 ± 15.54 × 21.53 ± 1.71	45.66 ± 1.31 × 28.77 ± 0.92	43.74 ± 1.38 × 26.80 ± 1.08	30.06	25.25		67.94 ± 5.79	55.64 ± 5.38	
*G. divaricata*	373.01 ± 14.24 × 25.19 ± 0.78	342.65 ± 11.71 × 25.45 ± 1.46	59.25 ± 1.36 × 31.47 ± 1.01	56.12 ± 1.65 × 26.87 ± 1.10	38.17	39.94		73.93 ± 11.55	113.66 ± 8.95	
*G. fedtschenkoana*	289.94 ± 14.17 × 30.10 ± 1.68	248.90 ± 9.50 × 28.78 ± 1.61	59.87 ± 1.42 × 33.62 ± 0.93	55.36 ± 1.70 × 36.79 ± 1.21	39.17	36.51		73.08 ± 6.40	78.52 ± 4.31	
*G. filiformis*	293.45 ± 23.42 × 23.09 ± 2.35	304.18 ± 27.82 × 26.30 ± 2.50	66.78 ± 2.21 × 33.30 ± 1.95	63.00 ± 1.89 × 28.76 ± 0.77	21.05	26.70		38.76 ± 7.72	52.24 ± 10.62	
*G. fragifera*	403.84 ± 42.01 × 29.10 ± 2.76	368.47 ± 26.21 × 25.55 ± 0.81	72.60 ± 1.16 × 33.78 ± 1.04	75.93 ± 1.02 × 35.68 ± 0.84	25.08	25.21		39.81 ± 3.74	37.97 ± 2.74	
*G. granulosa*	573.48 ± 17.45 × 37.39 ± 0.94	545.82 ± 52.66 × 50.03 ± 1.48	81.46 ± 2.15 × 41.44 ± 1.12	74.53 ± 1.93 × 35.54 ± 0.87	30.93	36.79		15.95 ± 3.10	19.90 ± 1.55	
*G. jaeschkei*	323.32 ± 19.56 × 33.01 ± 1.89	381.79 ± 27.95 × 35.33 ± 3.60	48.63 ± 0.87 × 28.95 ± 1.03	58.31 ± 2.01 × 35.95 ± 1.46	26.86	32.12		47.91 ± 12.91	39.04 ± 7.11	
*G. jensii*	287.58 ± 13.62 × 23.24 ± 1.17	281.01 ± 9.71 × 24.69 ± 1.49	59.23 ± 1.65 × 30.67 ± 1.41	56.73 ± 1.88 × 30.67 ± 1.31	25.38	35.74		46.15 ± 3.97	65.41 ± 10.40	
*G. nigra*	285.60 ± 9.59 × 24.68 ± 1.54	297.71 ± 19.87 × 28.01 ± 1.53	60.43 ± 2.03 × 35.72 ± 1.85	55.79 ± 1.16 × 32.65 ± 1.50	21.77	29.73		42.64 ± 7.14	51.65 ± 4.28	
*G. kunawurensis*	460.94 ± 8.64 × 30.28 ± 1.22	402.37 ± 15.31 × 2.57 ± 1.65	63.87 ± 1.19 × 32.82 ± 0.44	62.02 ± 1.92 × 32.32 ± 0.52	22.05	22.83		29.72 ± 2.33	27.31 ± 1.57	
*G. stepposa*	254.37 ± 10.33 × 23.08 ± 1.22	228.31 ± 7.42 × 24.95 ± 1.28	46.68 ± 1.94 × 25.96 ± 0.40	46.91 ± 0.71 × 29.33 ± 0.43	22.42	24.71		58.63 ± 6.06	58.81 ± 5.84	
*G. tenera*	474.34 ± 14.17 × 36.74 ± 3.24	505.18 ± 12.89 × 43.22 ± 4.07	82.16 ± 2.39 × 44.16 ± 0.77	92.82 ± 5.17 × 42.30 ± 1.42	26.01	31.54		31.86 ± 2.52	32.53 ± 7.04	

*Note*: Ad: adaxial; Ab: abaxial; L: length; W: width

### Morphology of basal leaf

Leaf morphological of the studied species showed two characters: eight species had cauline leaves (Fig. 1A-C, I, J, L-N) and six species did not (Fig. 1D-H, K). Length and width of basal leaf ranged from 54.12 ± 3.67 to 189.76 ± 15.49 mm and from 0.70 ± 0.04 to 4.65 ± 0.22 mm, respectively (Table 2). Longest basal leaf was found in *G. jensii*, whereas the shortest one was found in *G. bulbifera* (Table 2). Widest basal leaf was found in *G. granulosa*, whereas the narrowest one was found in *G. bulbifera* (Table 2). The basal leaf of *G. altaica* (Fig. 1B), *G. bulbifera* (Fig. 1C), *G. divaricata* (Fig. 1D), *G. filiformis* (Fig. 1F), *G. kunawurensis* (Fig. 1L) and *G. tenera* (Fig. 1N) was terete, while that of the other species was flat (Table 2). A transverse section of basal leaf was triangular (Fig. 1A1-D1, F1, J1 M1), flat (Fig. 1E1, H1, I1, K1) or circular (Fig. 1G1, L1, N1), or sometimes fistulose (Fig. 1J1).

### Morphology of leaf epidermis

Glandular trichomes were observed on both surfaces of *G. stepposa* (Fig. 2M, M1), while dendroid-type trichomes were found on the adaxial surface of *G. alberti* and *G. jensii* (Fig. 2A, J). Leaf epidermis was comprised of long and short cells, although not all species possessed short cells (Table 4), such as *G. filiformis* (Fig. 2F, F1), *G. fragifera* (Fig. 2G, G1), *G. kunawurensis* (Fig. 2L, L1), *G. stepposa* (Fig. 2M, M1) and *G. tenera* (Fig. 2N, N1). Long cells were parallel to the leaf veins and varied from rectangular (Fig. 2A, A1, B, B1, H, H1, J, J1, N, N1), rhomboid (Fig. 2C, C1, D, D1, G, G1, I, I1, M, M1) to irregular (Fig. 2E, E1, F, F1, L, L1). Pattern of anticlinal walls was either straight or sinuous (Table 3). The size of long cells was significantly different between species (H = 87.571, *df* = 13, *p* < 0.001). The largest epidermal cells were observed in *G. granulosa* (Adaxial: 545.82 ± 52.66 × 50.03 ± 1.48 μm, Abaxial: 573.48 ± 17.45 × 37.39 ± 0.94 μm) and smallest ones in *G. stepposa* (Adaxial: 228.31 ± 7.42 × 24.95 ± 1.28 μm, Abaxial: 254.37 ± 10.33 × 23.08 ± 1.22 μm). Examination of the epidermal cells on both surfaces of *Gagea* under LM revealed that their morphology was roughly identical (Tables 3 and 4).

### Morphology of stomata

Orientation of all stomata on the leaf epidermis of *Gagea* was roughly consistent (Fig. 2). All species have amphistomatic stomata, i.e. on both surfaces, that are paracytic (Fig. 2). Stomatal size (H = 91.717, *df* = 13, *p* < 0.001) and stomatal index (H = 93.654, *df* = 13, *p* < 0.001) differed significantly between species. Stomatal index (SI) ranged from 21.05 to 39.17% on adaxial surface in *G. filiformis* and *G. fedtschenkoana* respectively, whereas 22.83–39.94% on abaxial surface in *G. kunawurensis* and *G. divaricata* respectively (Table 4). Size of stomata ranged from 45.66 ± 1.31 × 28.77 ± 0.92 to 82.16 ± 2.39 × 44.16 ± 0.77 μm on adaxial surface of *G. bulbifera* and *G. tenera*, respectively, whereas on abaxial surface size ranged from 43.74 ± 1.38 × 26.80 ± 1.08 μm to 92.82 ± 5.17 × 42.30 ± 1.42 μm in *G. bulbifera* and *G. tenera*, respectively (Table 4).

### Hierarchical cluster analysis

**Fig. 3:**
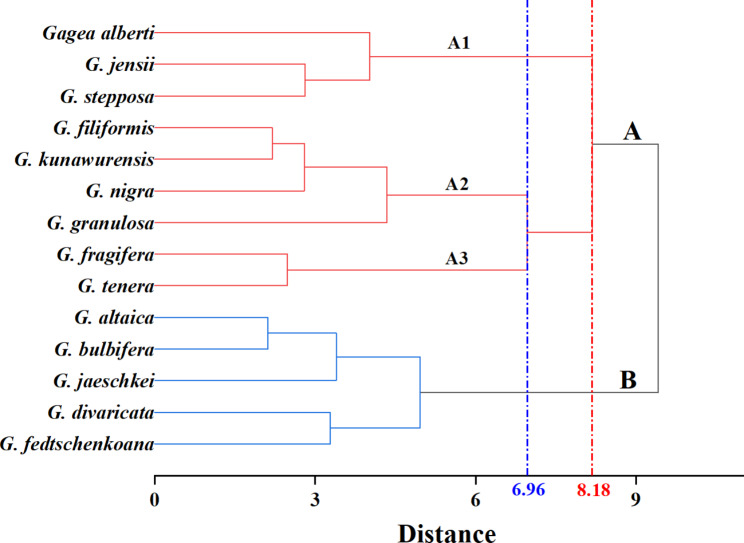
Cluster diagram (Ward’s linkage) of 14 species in *Gagea* from Xinjiang, China, based on six qualitative and three quantitative morphological and epidermal characters of leaves

A hierarchical clustering dendrogram shows the relationship between the 14 species based on leaf morphological and epidermal characters (Fig. 3). The obtained tree separated the species into two groups based on a Euclidean distance of 8.18. Group A was composed of nine species: *G. alberti*, *G. filiformis*, *G. fragifera*, *G. granulosa*, *G. jensii*, *G. kunawurensis*, *G. nigra*, *G. stepposa* and *G. tenera*. Further, Group A was arranged into three subgroups (A1, A2 and A3), based on a Euclidean distance of 6.96. Whereas group B included *G. altaica*, *G. bulbifera*, *G. divaricata*, *G. fedtschenkoana* and *G. jaeschkei* (Fig. 3).

## Discussion

The present study provides some baseline data about leaf morphology and leaf epidermal anatomy characters of *Gagea* from Xinjiang, China. Observations of the leaf morphology and epidermal characters of 14 species of *Gagea* revealed the following characters: (1) except for *G. fragifera* and *G. tenera*, the basal leaf of most species was solid; (2) the adaxial and abaxial epidermal cells were mostly quadrilateral but a few are irregular; (3) the leaf epidermal layer consists of long and short cells, but *G. filiformis*, *G. fragifera*, *G. kunawurensis*, *G. stepposa* and *G. tenera* do not have short cells; (4) the pattern of anticlinal walls was sinuous or straight; (5) orientations of all stomatal in the leaf epidermis are roughly identical; (6) the basal leaf of *G. alberti*, *G. jensii* and *G. stepposa* is hairy. The indumentum (hairiness) of basal leaf can be divided into two types: glandular trichomes found in *G. stepposa* and dendroid-type found in all the other species. The leaf epidermis of *Gagea* shows sinuous or straight patterns of anticlinal walls, rectangular, rhomboid or irregular shape of the long cells, and presence of long and short cells in some species, and these are key characters that can be used for classification and identification of *Gagea*. These findings emphasize the importance of leaf morphology and epidermal characters in the taxonomic study of *Gagea*.

Wang et al. (2013) reported differences in leaf epidermal characters, including the microstructure of epidermal cells and stomata for five species of *Gagea*, each represented by a single population in China. In our comparative study of the five species studied by Wang et al. (2013), we had total of 24 populations for the five species, and we found interspecific differences but no significant differences within and among conspecific populations. Additionally, Elwan (2008) also demonstrated interspecific differences in leaf epidermal characters. These findings reflect the stability of leaf epidermal morphology within populations of the same species. For monocotyledons, the Liliaceae differs considerably from the Asparagaceae and Poaceae in terms of leaf epidermal characters (Aliscioni et al. 2015; Meng et al. 2016; Chao et al. 2022). The types of anticlinal wall of *Fritillaria* (Wang et al. 2009) and *Lilium* (Hou et al. 2015) in the Liliaceae are straight or curve, and types of anticlinal walls observed in *Gagea* are consistent with these genera. However, in addition to rectangular or rhombic shapes of long cells, serrated cells also are present in *Fritillaria* and *Lilium*, but are not present in *Gagea* (Wang et al. 2009; Hou et al. 2015). The stomata of Liliaceae exhibit consistent orientation and shape, providing significant taxonomic value among monocotyledon (Wang et al. 2009; Doğu 2019).

Leaf epidermal characteristics within each section are generally highly consistent. For instance, in sect. *Didymobulbos*, the basal leaves are typically circular, flattened, or fistulose in transverse section, with 1–2 basal leaves present. Similarly, sect. *Plecostigma* has 1–2 basal leaves that are circular in transverse section, while sect. *Platyspermum* has a single basal leaf that is pentagonal in transverse section; sect. *Gagea* features 1–2 basal leaves that are flattened in transverse section (Zarrei et al. 2010, 2011). Our research corroborates these results, demonstrating that the consistency in leaf epidermal characteristics within each section yields valuable insights for the classification and identification of *Gagea*.

Currently, classification studies of most species are conducted from a molecular perspective, which can achieve classification results at the genetic level. However, compared to external macroscopic morphological characters, there are limitations in practical species identification (Shneyer et al. 2003; Hou et al. 2015). Morphologically, the *Gagea* species are discriminated using information on bulbs, leaves, inflorescences, tepals, stigmas and seeds traits (Chen and Turland 2000). The level of variation in anatomical characters is the highest among the basal leaf (Zarrei et al. 2010). In contrast, classification based on leaf morphology and epidermal characters is a relatively intuitive and practical method. Species with similar external morphology can be inferred from leaf morphological and epidermal characters, which have been reported in *Ornithogalum* (Peruzzi et al. 2007), *Fritillaria* (Wang et al. 2009), *Maianthemum* (Meng et al. 2016), *Impatiens* (Rahman et al. 2017), *Aspidistra* (Vislobokov et al. 2021) and *Zingiber* (Zhao et al. 2022). An extensive field investigation and examination of specimens from Xinjiang found that *G. nigra* and *G. filiformis*; *G. jensii*, *G. altaica* and *G. alberti* have similar morphological characters, such as bulbs, inflorescences, tepals, stigmas and seeds. Thus, these species are closely related natural taxa and support their placement in the same section (Tison et al. 2013; Peterson et al. 2016). The absence of short cells in *G. filiformis* (Fig. 2F, F1) can be used to distinguish this species from *G. nigra* (Fig. 2K, K1). The leaf morphology is consistent with Peterson et al.’s (2016) classification of *G. nigra*. The sinuous pattern of anticlinal walls of *G. altaica* (Fig. 2B, B1) also can be used to separate this species from *G. jensii* (Fig. 2J, J1) and *G. alberti* (Fig. 2A, A1). Remaining species also can be distinguished based on their pollen morphology, except for *G. jensii* (Lin et al. 2023). Morphologically, *G. jensii* and *G. alberti* exhibit slight differences; *G. jensii* has a denser inflorescence and larger lateral flowers, as stated by Peterson et al. (2011). Nonetheless, it is difficult to distinguish two species based solely on pollen grains (Lin et al. 2023) and leaf epidermis, and employment of cytological or molecular evidence to identify *G. jensii*, is necessary. Classification research based on leaf and epidermal characters can not only reflect common characters of *Gagea* but also interspecific variability. Therefore, leaf morphology and epidermal characters are considered highly valuable for the systematic classification of *Gagea*.

## Conclusion

This study is the first to combine the leaf structure of *Gagea* with detailed epidermal characters. While not all variable traits hold systematic or taxonomic significance, a considerable number of them play a crucial role in species differentiation, such as the shape of leaf epidermal long cells. The division of species with similar external morphology is further supported by phylogenetic and palynological evidence. The leaf and its epidermal characters provide evidence for species classification, with significant differences in epidermal characters that can help resolve delimitation of species with similar appearances in *Gagea*. Consequently, these characters hold systematic significance and enhance our understanding of *Gagea*.

### Electronic supplementary material

Below is the link to the electronic supplementary material.

**Supplementary Material: Table S1**. The list of plant materials examined and vouchers of 50 populations belonging to 14 species of Gagea from Xinjiang, China

## Data Availability

All data generated or analyzed during this study are included in this published article and its additional files.
