# Supplementary material for: Taxonomic implications of leaf morphology and epidermal anatomy for 14 species of Gagea (Liliaceae) from Xinjiang, China
Source: Bot Stud. 2023 Nov 29;64:33. doi: 10.1186/s40529-023-00405-9 (PMC10684841; doi:10.1186/s40529-023-00405-9)
Supplement: Supplementary file 1 — Supplementary Material: Table S1. The list of plant materials examined and vouchers of 50 populations belonging to 14 species of Gagea from Xinjiang, China [file 40529_2023_405_MOESM1_ESM.docx]

The list of plant materials examined and vouchers of 50 populations belonging to 14 species of *Gagea* from Xinjiang, China.

| Species | Locality | Coordinate | Altitude | Collection Date | Voucher |
| --- | --- | --- | --- | --- | --- |
| *Gagea alberti* (1) | Shihezi City, Xinjiang, China | 44.188091°N, 86.088827°E | 517 m | 12 April 2022 | J.Qiu & D.Y.Tan  L-034 (XJA) |
| *G. alberti* (2) | Urumqi City, Xinjiang, China | 43.857741°N, 87.544662°E | 795 m | 16 April 2022 | J.Qiu & D.Y.Tan  L-039 (XJA) |
| *G. alberti* (3) | Huocheng County, Xinjiang, China | 44.004373°N, 80.936525°E | 599 m | 21 April 2022 | J.Qiu & D.Y.Tan  L-054 (XJA) |
| *G. alberti* (4) | Yining City, Xinjiang, China | 43.724521°N, 82.070256°E | 1033 m | 22 April 2022 | J.Qiu & D.Y.Tan  L-062 (XJA) |
| *G. altaica* (1) | Fuyun County, Xinjiang, China | 46.368831°N, 88.926181°E | 777 m | 15 April 2021 | J.Qiu & M.S.Lin  L-006 (XJA) |
| *G. altaica* (2) | Fuyun County, Xinjiang, China | 46.645528°N, 88.593935°E | 793m | 16 April 2021 | J.Qiu & M.S.Lin  L-007 (XJA) |
| *G. altaica* (3) | Fuyun County, Xinjiang, China | 46.830949°N, 88.791038°E | 703 m | 16 April 2021 | J.Qiu & M.S.Lin  L-009 (XJA) |
| *G. bulbifera* (1) | Shawan City, Xinjiang, China | 45.328851°N, 88.455975°E | 561 m | 7 April 2021 | J.Qiu & M.S.Lin  L-002 (XJA) |
| *G. bulbifera* (2) | Fuyun County, Xinjiang, China | 46.841351°N, 89.405816°E | 1028 m | 16 April 2021 | J.Qiu & M.S.Lin  L-008 (XJA) |
| *G. bulbifera* (3) | Urumqi City, Xinjiang, China | 43.830091°N, 87.780818°E | 996 m | 18 April 2021 | J.Qiu & M.S.Lin  L-013 (XJA) |
| *G. bulbifera* (4) | Shihezi City, Xinjiang, China | 44.194476°N, 86.079483°E | 664 m | 12 April 2022 | J.Qiu & D.Y.Tan  L-033 (XJA) |
| *G. bulbifera* (5) | Huocheng County, Xinjiang, China | 44.004373°N, 80.936525°E | 599 m | 21 April 2022 | J.Qiu & D.Y.Tan  L-055 (XJA) |
| *G. bulbifera* (6) | Yining City, Xinjiang, China | 43.817749°N, 81.902141°E | 880 m | 22 April 2022 | J.Qiu & D.Y.Tan  L-058 (XJA) |
| *G. divaricata* (1) | Fuhai County, Xinjiang, China | 45.053634°N, 88.398225°E | 712 m | 17 April 2021 | J.Qiu & M.S.Lin  L-010 (XJA) |
| *G. divaricata* (2) | Fukang City, Xinjiang, China | 44.739223°N, 88.270266°E | 616m | 17 April 2021 | J.Qiu & M.S.Lin  L-011 (XJA) |
| *G. divaricata* (3) | Huocheng County, Xinjiang, China | 44.018193°N, 80.775512°E | 607 m | 21 April 2022 | J.Qiu & D.Y.Tan  L-056 (XJA) |
| *G. fedtschenkoana* (1) | Nilka County, Xinjiang, China | 43.652369°N,  84.362144°E | 2278 m | 2 June 2020 | J.Qiu & D.Y.Tan  NLK-003 (XJA) |
| *G. fedtschenkoana* (2) | Bole City, Xinjiang, China | 44.632994°N,  81.329544°E | 2091 m | 3 June 2020 | J.Qiu & D.Y.Tan  SH-001 (XJA) |
| *G. fedtschenkoana* (3) | Qinghe County, Xinjiang, China | 46.746946°N,  90.873269°E | 2761 m | 6 June 2021 | J.Qiu & M.S.Lin  L-018 (XJA) |
| *G. fedtschenkoana* (4) | Burqin County, Xinjiang, China | 48.504169°N,87.138269°E | 1441 m | 9 June 2021 | J.Qiu & M.S.Lin  L-022 (XJA) |
| *G. fedtschenkoana* (5) | Hutubi County County, Xinjiang, China | 43.880653°N, 86.480528°E | 1151 m | 20 April 2022 | J.Qiu & D.Y.Tan  L-045 (XJA) |
| *G. filiformis* (1) | Urumqi City, Xinjiang, China | 43.786772°N, 87.565508°E | 1075 m | 3 April 2021 | J.Qiu & M.S.Lin  L-004 (XJA) |
| *G. filiformis* (2) | Yuming County, Xinjiang, China | 45.842106°N, 82.525295°E | 1676 m | 28 April 2021 | J.Qiu & D.Y.Tan  Yu-002 (XJA) |
| *G. filiformis* (3) | Burqin County, Xinjiang, China | 48.430501°N, 87.210654°E | 1987 m | 9 June 2021 | J.Qiu & M.S.Lin  L-025 (XJA) |
| *G. filiformis* (4) | Manas County, Xinjiang, China | 43.865031°N, 86.252183°E | 1526 m | 20 April 2022 | J.Qiu & D.Y.Tan  L-049 (XJA) |
| *G. fragifera* (1) | Qinghe County, Xinjiang, China | 46.781335°N,90.887534°E | 2662 m | 6 June 2021 | J.Qiu & M.S.Lin  L-017 (XJA) |
| *G. fragifera* (2) | Fuhai County, Xinjiang, China | 48.059008°N, 88.920102°E | 2401 m | 7 April 2021 | J.Qiu & M.S.Lin  L-019 (XJA) |
| *G. fragifera* (3) | Burqin County, Xinjiang, China | 48.429559°N,87.207309°E | 1988 m | 9 June 2021 | J.Qiu & M.S.Lin  L-021 (XJA) |
| *G. granulosa* (1) | Xinyuan County, Xinjiang, China | 43.248167°N, 84.011416°E | 1882 m | 4 June 2020 | J.Qiu & D.Y.Tan  XY-002 (XJA) |
| *G. granulosa* (2) | Burqin County, Xinjiang, China | 48.429694°N,87.207108°E | 1984 m | 9 June 2021 | J.Qiu & M.S.Lin  L-023 (XJA) |
| *G. granulosa* (3) | Yuming County, Xinjiang, China | 46.191895°N, 82.936688°E | 709 m | 28 April 2021 | J.Qiu & D.Y.Tan  Yu-004 (XJA) |
| *G. jaeschkei* (1) | Tekes County, Xinjiang, China | 43.411647°N,81.040648°E | 2538 m | 9 June 2020 | J.Qiu & D.Y.Tan  TKS-003 (XJA) |
| *G. jaeschkei* (2) | Qapqal County, Xinjiang, China | 42.993913°N, 82.213955°E | 2929 m | 17 July 2021 | J.Qiu & M.S.Lin  L-30 (XJA) |
| *G. jaeschkei* (3) | Urumqi City, Xinjiang, China | 43.119812°N,86.855626°E | 3492 m | 6 June 2022 | M.S.Lin & D.Y.Tan  L-069 (XJA) |
| *G. jaeschkei* (4) | Bole City, Xinjiang, China | 44.519168°N, 81.249635°E | 2095 m | 24 June 2022 | J.Qiu & D.Y.Tan  L-070 (XJA) |
| *G. jensii* | Urumqi City, Xinjiang, China | 43.783443°N, 87.544818°E | 1002 m | 8 April 2021 | J.Qiu & M.S.Lin  L-005 (XJA) |
| *G. nigra* (1) | Urumqi City, Xinjiang, China | 43.783141°N, 87.544363°E | 995 m | 2 April 2021 | J.Qiu & M.S.Lin  L-003 (XJA) |
| *G. nigra* (2) | Urumqi City, Xinjiang, China | 43.813774°N, 88.994927°E | 1837 m | 18 April 2021 | J.Qiu & M.S.Lin  L-012 (XJA) |
| *G. nigra* (3) | Xinyuan County, Xinjiang, China | 43.778801°N, 83.456579°E | 1400 m | 9 April 2021 | X.J.Shi & X.Jiang  L-027 (XJA) |
| *G. nigra* (4) | Burqin County, Xinjiang, China | 48.430593°N, 87.210654°E | 1988 m | 9 June 2021 | J.Qiu & M.S.Lin  L-024 (XJA) |
| *G. nigra* (5) | Yuming County, Xinjiang, China | 45.842106°N, 82.525295°E | 1676 m | 28 April 2021 | J.Qiu & D.Y.Tan  Yu-001 (XJA) |
| *G. nigra* (6) | Yuming County, Xinjiang, China | 45.976943°N, 82.886943°E | 1908 m | 28 April 2021 | J.Qiu & D.Y.Tan  Yu-008 (XJA) |
| *G. kunawurensis* (1) | Urumqi City, Xinjiang, China | 43.785813°N, 87.545323°E | 997 m | 29 April 2021 | J.Qiu & M.S.Lin  L-015 (XJA) |
| *G. kunawurensis* (2) | Shihezi City, Xinjiang, China | 44.194476°N, 86.079483°E | 664m | 12 April 2022 | J.Qiu & D.Y.Tan  L-035 (XJA) |
| *G. kunawurensis* (3) | Shawan City, Xinjiang, China | 44.235896°N, 85.821398°E | 903 m | 20 April 2022 | J.Qiu & D.Y.Tan  L-052 (XJA) |
| *G. kunawurensis* (4) | Yining City, Xinjiang, China | 43.817749°N, 81.902141°E | 880 m | 22 April 2022 | J.Qiu & D.Y.Tan  L-057 (XJA) |
| *G. stepposa* (1) | Urumqi County, Xinjiang, China | 43.516102°N, 87.447984°E | 1559 m | 10 April 2022 | J.Qiu & D.Y.Tan  L-032 (XJA) |
| *G. stepposa* (2) | Hutubi County, Xinjiang, China | 43.821214°N, 86.429608°E | 1160 m | 20 April 2022 | J.Qiu & D.Y.Tan  L-040 (XJA) |
| *G. tenera* (1) | Yining City, Xinjiang, China | 43.625463°N, 82.128763°E | 600 m | 29 April 2020 | J.C.Chi  Chijc1907 (XJA) |
| *G. tenera* (2) | Nilka County, Xinjiang, China | 43.724538°N, 82.070252°E | 1033 m | 22 April 2022 | J.Qiu & D.Y.Tan  L-041 (XJA) |
